# The aid of “bedside ultrasonography” for the emergency surgeon: the experience of a single centre

**DOI:** 10.1186/2036-7902-6-S2-A3

**Published:** 2014-08-27

**Authors:** Antonino Agrusa, Giorgio Romano, Giuseppe Frazzetta, Giuseppe Amato, Daniela Chianetta, Silvia Di Giovanni, Giuseppe De Vita, Giuseppe Di Buono, Vincenzo Sorce, Gaspare Gulotta

**Affiliations:** 1 Dipartimento di Chirurgia Generale e D’Urgenza e dei Trapianti D’Organo, Azienda Ospedaliera Universitaria Policlinico “Paolo Giaccone” Palermo, Via Liborio Giuffrè 28 90100 Palermo, Sicilia, Italy

## Introduction

Abdominal blunt traumas are about 8-10% of all causes of death for trauma, with an incidence only slightly less than head trauma [1]; most of them are due to roads crashes. In polytraumatized patients, echographic examination with Focused Assessment with Sonography in Trauma is often the first approach that can give useful indications and informations for the therapeutic strategy [2].

## Aim

We performed a retrospective study on the use of ultrasounds, according to FAST protocol, for evaluation and monitoring of polytraumatized patients, admitted to our institution with a blunt abdominal trauma [3]. Our aim is to evaluate efficacy and diagnostic improvement of “Bedside Ultrasonography” in surgeon’s hands and to evaluate its capacity in driving therapeutic choices.

## Materials and methods

We examined the period between January 2012 and December 2013, selecting 33 patients come to our attention, in the department of emergency surgery in Policlinico of Palermo with polytrauma or blunt abdominal trauma, classified on triage as Class I or II (emergency or urgency): 26 with road trauma injuries; 7 with other causes.

## Results

Five patients (15%) were excluded from the study because of neurosurgical and/or orthopedic complications, having no abdominal injuries, documented by echography or CT. 16 of the remaining 28 patients (48, 5%) had instable hemodynamic conditions so they underwent to explorative laparotomy and splenectomy for spleen rupture (13 patients – 81%) or major liver damage (3 patients – 19%), documented by CT or FAST performed in emergency room. For 12 patients (36, 4%) with stable hemodynamic conditions when admitted, it was considered a conservative treatment, so, in the hours after, they underwent to “bedside ultrasonography” for: retroperitoneal hematoma (2 patients – 17%), undergoing, when the lesion became stable, to CT-guided drainage. Respectively 3 patients (25%) and 4 patients (33, 3%) had hepatic and renal minor injuries and underwent to non-operative management and echographic follow-up; 3 patients (25%) were followed with echographic approach because of “heterogeneous sonographic appearance of splenic parenchyma” and subsequent delayed spleen rupture. These patients underwent to surgery after echographic demonstration of sudden increase of abdominal effusion and signs of hypovolemic shock.

## Conclusions

Our experience confirms the usefulness of "bedside" ultrasound technique as indispensable tool in the skills of the emergency surgeon [4]. Speed, feasibility, easy repeatability several in time, lack of invasiveness avoid delays and reduce diagnostic errors, driving therapeutic choices of the surgeon, in agreement and sometimes in lieu of laboratory data [5].
